# Does arthroscopic or open washout in native knee septic arthritis result in superior post-operative function? A systematic review and meta-analysis of randomised controlled trials and observational studies

**DOI:** 10.1186/s13643-024-02508-1

**Published:** 2024-04-12

**Authors:** Grace E. M. Kennedy, Abisha Tharmaseelan, Jonathan R. A. Phillips, Jon T. Evans, Setor K. Kunutsor

**Affiliations:** 1 Royal Devon University Healthcare NHS Foundation Trust, Exeter, UK; 2 Bedfordshire Hospitals NHS Foundation Trust, Bedford, UK; 3 University of Exeter, Exeter, UK; 4 University of Leicester, Leicester, UK

## Abstract

**Aims:**

Septic arthritis (SA) of the native knee joint is associated with significant morbidity. This review compared post-operative functional outcomes (patient-reported outcome measures (PROMs) and range of movement (ROM)) following arthroscopic washout (AW) and open washout (OW) amongst adult patients with SA of the native knee. The need for further operative intervention was also considered.

**Methods:**

Electronic databases of PubMed, MEDLINE, Embase, Cochrane, Web of Science and Scopus were searched between 16 February 2023 and 18 March 2023. Randomised controlled trials (RCTs) and comparative observational analytic studies comparing function (reflected in PROMs or ROM) at latest follow-up following AW and OW were included. A narrative summary was provided concerning post-operative PROMs. Pooled estimates for mean ROM and re-operation rates were conducted using the random-effects model. The risk of bias was assessed using the Cochrane risk-of-bias assessment tool-2 for RCTs and the Risk of Bias in Non-Randomized Studies of Interventions tool for observational analytic studies.

**Results:**

Of 2580 retrieved citations, 7 articles (1 RCT and 6 cohort studies) met the inclusion criteria. Of these, five had some concerns/moderate risk of bias, and two had serious risk. There was a slight tendency for superior mean PROMs following AW compared with OW, but due to small effect sizes, this was unlikely clinically relevant. Additionally, the use of four different PROMs scales made direct comparisons impossible. AW was associated with superior ROM (mean difference 20.18° (95% *CI* 14.35, 26.02; *p* < 0.00001)), whilst there was a tendency for lower re-operation requirements following AW (*OR* 0.64, 95% *CI* 0.26, 1.57, *p* = 0.44).

**Conclusions:**

AW was associated with equivalent to superior post-operative function and lower requirement for further intervention compared with OW. Results need to be interpreted cautiously, taking into consideration the methodological and clinical heterogeneity of the included studies.

**Systematic review registration:**

PROSPERO 2022, CRD42022364062.

## Introduction

Septic arthritis (SA) of the native knee joint is an orthopaedic emergency, with treatment delays potentially resulting in significant cartilage disruption, or even life-threatening sepsis [1–3]. The incidence is approximately 2–10 per 100,000 persons in the UK [3, 4]. Risk factors include rheumatoid arthritis, skin infections, increasing age, bacteraemia, diabetes mellitus, liver disease, immunosuppression, and joint penetration [5, 6]. In adults, typical micro-organisms include *Staphylococcus aureus* (*S. aureus*) and streptococci [3, 4, 7].

Diagnosis of SA requires consideration of clinical and laboratory features. Patients typically report knee pain, swelling, erythema, restricted range of movement, and decreased weight bearing [6]. White blood cell counts and C-reactive protein levels may be elevated, whilst joint fluid aspirate reveals a causative micro-organism in approximately 50–75% cases [3, 6]. Culture-negative SA may arise due to sampling after antimicrobial therapy, rare micro-organisms not grown on regular culture media, and other technical factors [8–10]. Such absence of micro-organisms may be falsely reassuring, delaying treatment, and hindering ability to target antimicrobial therapy [10].

Management of native knee SA typically involves irrigation and debridement of the joint, commonly known as a ‘washout’. This can be performed arthroscopically (‘keyhole’) or via arthrotomy (‘open’) [6]. Removal of the synovial lining of the joint, synovectomy, may be undertaken as part of an arthroscopic or open washout and is thought to maximise the reduction of the bacterial burden, although the evidence for this is limited [11]. Antimicrobial therapy typically is recommended for up to 6-week post-washout [12] but may vary according to clinical and microbiological findings.

To our knowledge, no systematic review has compared post-operative function (reflected in patient-reported outcome measures (PROMs) and range of movement (ROM)) as a primary outcome following arthroscopic washout (AW) or open arthrotomy washout (OW) of native knee SA. Additionally, the literature varies regarding requirement for subsequent intervention, a potential complication of both AW and OW. Two recent meta-analyses explored this as their primary outcome [13, 14]. Liang found that AW and OW were associated with comparable rates of reinfection (odds ratio (OR) = 0.85) [13], whilst Panjwani et al. reported a lower pooled relative risk (RR) of reoperation following AW (*RR* = 0.69) [14].

We hypothesised that AW would be associated with favourable post-operative PROMs and ROM, owing to smaller incisions and reduced scarring. We also hypothesised that AW would be associated with as good, or superior, rates of infection eradication, in keeping with previous meta-analyses [13, 14]. Therefore, the primary aim of this review was to compare post-operative function following AW and OW. Secondary aims were to compare rates of reoperation in the early post-operative phase (30 days) and following typical cessation of antimicrobial therapy (90 days).

## Materials and methods

### Data sources and study selection

This systematic review was registered with PROSPERO (CRD42022364062) and was conducted based on a predefined protocol and in accordance with Preferred Reporting Items for Systematic Reviews and Meta-Analyses (PRISMA) guidelines [15].

We searched for studies that compared functional outcomes following AW and OW as the index procedure for native knee SA in adult patients (> 18 years).

The online databases PubMed, MEDLINE, Embase, Cochrane, Web of Science, and Scopus through OvidSP were searched independently by two authors (G. K., A. T.) between 16 February 2023–18 March 2023, according to the agreed search strategies, using combined text and MeSH headings (Table 1). Databases were searched from database inception with no date range imposed on the retrieval of studies.

**Table 1:** Search strategies as devised for each of the searched databases

Database	Search strategy
PubMed	(Septic Arthritis[tiab] OR Suppurative Arthritis[tiab] OR infect* Arthritis[tiab] OR Pyogenic Arthritis[tiab] OR Bacterial Arthritis[tiab] OR Arthritis, Infectious[MeSH]) AND (Arthrotomy[tiab] OR Open[tiab] OR Arthroscop*[tiab] OR Arthroscopy[MeSH]) AND (Knee*[tiab] OR Knee Joint[MeSH] OR Knee[MeSH])
MEDLINE (1946 onwards)	#1(Septic Arthritis or Suppurative Arthritis or infect* Arthritis or Pyogenic Arthritis or Bacterial Arthritis).mp
	#2infectious arthritis.mp. or exp *Arthritis, Infectious/
	#3(Arthroscop* or Arthrotomy or Open).mp
	#4arthroscopy.mp. or exp *Arthroscopy/
	#5exp *Knee/ or exp *Knee Joint/ or knee.mp
	#61 or 2
	#73 or 4
	#85 and 6 and 7
Embase (1980 onwards)	#1(Septic Arthritis or Suppurative Arthritis or infect* Arthritis or Pyogenic Arthritis or Bacterial Arthritis).mp
	#2limit 1 to abstracts
	#3exp *infectious arthritis/
	#4(Arthroscop* or Arthrotomy or Open).mp
	#5limit 4 to abstracts
	#6exp *arthroscopy/
	#7Knee.mp. or exp *knee/
	#82 or 3
	#95 or 6
	#107 and 8 and 9
Cochrane	#1Septic Arthritis OR Suppurative Arthritis OR infect* Arthritis OR Pyogenic Arthritis OR Bacterial Arthritis:ti,ab,kw
	#2Arthritis, Infectious
	#3Arthroscop* OR Arthrotomy OR Open:ti,ab,kw
	#4Arthroscopy
	#5Knee*:ti,ab,kw
	#6Knee37105
	#7Knee joint
	#8#1 OR #2
	#9#3 OR #4
	#10#5 OR #6
	#11#7 AND #8 AND #9
Web of Science (1900 onwards)	#1 ((((AB = (septic arthritis)) OR AB = (suppurative arthritis)) OR AB = (infectious arthritis)) OR AB = (pyogenic arthritis)) OR AB = (bacterial arthritis)
	#2 (((AB = (arthrotomy)) OR AB = (open)) OR AB = (arthrosc*)) OR AB = (arthroscopy)
	#3 (AB = (knee)) OR AB = (knee joint)
	#4 #1 AND #2 AND #3
Scopus	TITLE-ABS-KEY ( ( septic AND arthritis OR suppurative AND arthritis OR infect* AND arthritis OR pyogenic AND arthritis OR bacterial AND arthritis OR arthritis, AND infectious) AND ( arthrotomy OR open OR arthroscop* OR arthroscopy) AND ( knee* OR knee AND joint OR knee))

Article titles and abstracts, and then full manuscripts of potentially relevant studies, were independently reviewed by two authors (G. K., A. T.) who discussed and resolved any disagreements regarding inclusion, without needing to consult the senior authors (S. K., J. E.). The reference lists of relevant publications were also hand-searched for additional relevant studies.

Studies were included if they were interventional or comparative observational analytic studies (randomised controlled trials (RCTs), cohort studies, case–control studies) involving human subjects. We excluded narrative reviews, case reports, letters to the editor, and studies describing prosthetic joint infections or noninfectious arthritis.

### Data extraction

One author (G. K.) used a standardised form to extract data. A second reviewer (A. T.) independently checked these data against those in original articles.

Data were extracted on the following: geographical location, publication year, study design, level of evidence [16], participants (age, sex), sample size, duration of follow-up, risk factors, microbiological findings, post-operative PROMs and ROM, re-operation requirements, and synovectomy at index procedure.

In publications where data were inadequate, we contacted the authors to request the information needed. Where no response was obtained, the study was excluded from analysis.

### Outcomes

The primary outcomes were post-operative PROMs and ROM (at latest follow-up) following AW and OW. The secondary outcomes were rates of reoperation for persistent or recurrent infection within 30 and 90 days of index procedure. We also aimed to present an overview of microbiological findings, risk factors, and whether synovectomy was undertaken during the index procedure.

### Assessment of risk of bias and evidence quality

The risk of bias was independently assessed by two authors (G. K., A. T.) who discussed and resolved any disagreements. The Cochrane risk-of-bias assessment tool-2 (RoB2) [17] was used for RCTs and the Risk of Bias in Non-Randomized Studies of Interventions (ROBINS-I) tool [18] for observational studies.

The Grading of Recommendations Assessment, Development and Evaluation (GRADE) criteria were used to assess the quality of the evidence for each outcome [19].

### Data synthesis and analysis

A narrative summary was provided concerning PROMs, microbiological features, risk factors, and undertaking of synovectomy. Regarding ROM and reoperation, summary measures were presented as mean differences and odds ratios (OR) with 95% confidence intervals (95% *CI*). The random-effects model was used to obtain pooled estimates for each outcome, to account for interstudy heterogeneity and provide a more conservative evaluation of the significance of the association [20]. The extent of interstudy heterogeneity was assessed with the *I*^2^ statistic [21], with values of 30–60% representing moderate heterogeneity [22].

Statistical analysis was conducted using Review Manager (RevMan Web), version 5.4, the Cochrane Collaboration 2020, available at revman.cochrane.org.

## Results

### Article selection

**Fig. 1:**
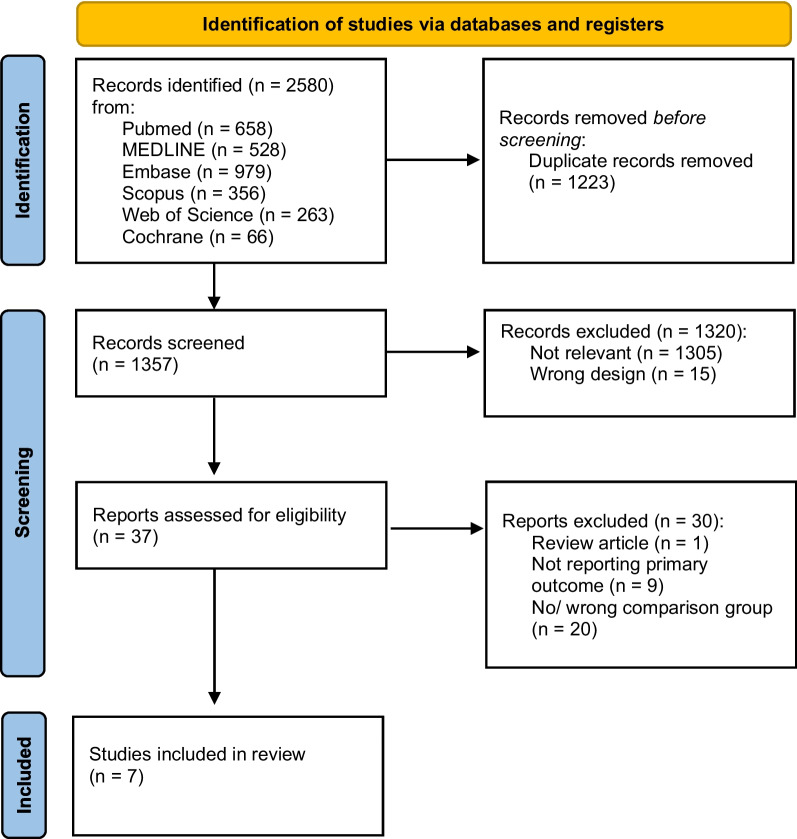
PRISMA flow chart outlining the study selection process

In total, 2580 potentially relevant citations were identified, 2573 of which were subsequently excluded (Fig. 1). Seven eligible studies were included.

### Study characteristics

Table 2 outlines characteristics of the seven studies (one RCT, six cohort studies) reporting functional outcomes after AW and OW. In total, 394 patients (243 arthroscopic, 151 open) were included.

**Table 2:** Characteristics of included studies

Study	Location	Study design	Evidence level^(16)^	Number of patients		Male (%)		Age (years)		Follow-up (months)
				AW	OW	AW	OW	AW	OW	
Peres (2016) [23]	Brazil	RCT	1	10	11	7 (70.0)	7 (63.6)	42 (11–66)^¶^	45.4 (19–74)^¶^	Min 24
Balabaud (2007 [24]	France	Cohort	3	21	19	31 (77.5)	31 (77.5)	49 ± 20 (19–81)^¶^		22 ± 2 (12–96)^¶^
Böhler (2016) [25]	Austria	Cohort	3	41	29	27 (65.9)	19 (65.5)	49 (30–64)¥	71 (65–78)¥	12¥ (12–15)
Johns (2017) [26]	Australia	Cohort	3	119	42	80 (67.2)	28 (66.7)	57.5 (15.8)^¥^	65.8 (16.0)^¥^	-
Kalem (2018) [27]	Turkey	Cohort	3	13	11	8 (61.5)	6 (54.5)	56.6 ± 14.9^¶^	59.5 ± 17.2^¶^	6
Sabater-Martos (2021) [28]	Spain	Cohort	3	12	15	18 (66.7)		64.8 (30–89)		Min 12
Wirtz (2001) [29]	Germany	Cohort	3	27	24	25 (49.0)		59.7 (21–94)^¶^		26.4^¶^

^¶^ Mean value ± standard deviation (range). ^¥^Median value (interquartile range)

Key microbiological findings are outlined in Table 3. *Staphylococcus aureus* was the most common micro-organism (96, 24.4%), whilst over 15% were culture negative (66, 16.5%). Where described, antimicrobial regimes were typically administered for a total of 4–6 weeks [23–27, 29]. No risk factors were present in at least 24.4% of patients (Table 3).

**Table 3:** Key microbiological findings and the presence of risk factors for SA development described in each study

Study	Microbiological diagnosis		Presence of risk factors	
	AW	OW	AW	OW
Peres [23]	Negative 11 (52.4%)		Idiopathic 10 (47.6%)	
			Chronic renal failure (CRF) 3 (14.3%)	
	*S. aureus* 6 (28.7%)		Repetition arthrocentesis 5 (23.8%)	
	*S. epidermidis* 2 (9.5%)		Pyoderma 2 (9.5%)	
	*S. agalactiae* 1 (4.7%)			
	*Klebsiella* species 1 (4.7%)			
Balabaud [24]	Negative 3 (7.5%)		Idiopathic 29 (72.5%)	
	Methicillin-sensitive *S. aureus* (MSSA) 12 (30.0%)		Diabetes mellitus (DM) 4 (10.0%)	
			Alcohol abuse 5 (12.5%)	
	Methicillin-resistant *S. aureus* (MRSA) 4 (10.0%)		CRF 1 (2.5%)	
			Psoriasis 1 (12.5%)	
	*S. epidermidis* 7 (17.5%)			
	Other staphylococci 7 (17.5%)			
	Gram-negative pathogens 4 (10.0%)			
Böhler [25]	–		Rheumatoid arthritis (RA) 4 (9.8%) DM 7 (17.1%)	RA 0 DM 10 (34.5%)
Johns [26]	Negative 23 (19.3%)	Negative 4 (9.5%) MSSA 16 (38.1%) MRSA 1 (2.4%) Streptococci 11 (26.2%) Gram negative 5 (11.9%)	None 45 (37.8%) DM 15 (12.6%) Liver disease 14 (11.8%) Intravenous drug use (IVDU) 11 (9.2%) CRF 14 (11.8%) RA 8 (6.7%)	None 10 (23.8%) DM 8 (19.0%) Liver disease 8 (19.0%) IVDU 6 (14.3%) CRF 3 (7.1%) RA (1 (2.4%)
	MSSA 41 (34.5%) MRSA 4 (3.4%) Streptococci 18 (15.1%)			
	Gram negative 8 (6.7%)			
Kalem [27]	MSSA 1 (7.7%)	MSSA 1 (9.1%)	DM 4 (30.8%)	DM 5 (45.5%)
		MRSA 2 (18.2%)	IVDU 3 (23.1%)	
			Liver disease 0 RA 1 (7.7%)	
				IVDU 0 Liver disease 2 (18.2%)
				RA 1 (9.1%)
Sabater-Martos [28]	Negative 4 (33.3%)	Negative 8 (53.3%)	American Society of Anaesthesiologists (ASA) I 2 (16.7%)	ASA I 0
				ASA II 9 (60.0%)
	*S. aureus* 4 (33.3%)	*S. aureus* 4 (2.7%)		
				ASA III 6 (40.0%)
			ASA II 4 (33.3%)	
			ASA III 6 (50.0%)	
	*S. epidermidis* 1 (8.3%)	*S. epidermidis* 1 (6.7%)		
	*Streptococcus* 2 (16.7%)	*Streptococcus* 2 (13.3%)		
Wirtz [29]	Negative 13 (25.5%)		–	
	Positive 38 (74.5%) (most often SA)			

Regarding disease severity, of the three studies [24, 26, 29] reporting Gächter stage [30], there was a tendency for patients with earlier changes (stages I/II) to be managed arthroscopically and more advanced changes (III/IV) to be managed with OW.

### Assessment of risk of bias

Moderate risk of bias was present in five studies and serious risk in two studies (Table 4). Bias in participant selection was mostly considered moderate because there may have been an association between the interventions and outcomes (patients with more severe symptoms were more likely to undergo OW). Bias relating to measurement of interventions, outcomes, and departures from intended interventions was judged low because the intervention and outcomes were objective and insusceptible.

**Table 4:** Risk-of-bias assessment of the randomised controlled trial by the RoB-2 assessment tool and of the cohort studies by ROBINS-1

Study								
	Bias arising from the randomisation process			Bias due to departures from intended interventions	Bias due to missing data	Bias in measurement of outcomes	Bias in selection of reported results	Overall bias
RoB-2 [17]								
Peres [23]	Low			Low	Low	Some concerns	Low	Some concerns
ROBINS-1 [18]	Bias due to confounding	Bias in selection of participants	Bias in measurement of interventions					
Balabaud [24]	Moderate	Moderate	Low	Low	Low	Low	Low	Moderate
Böhler [25]	Moderate	Moderate	Low	Low	Low	Low	Low	Moderate
Johns [26]	Moderate	Moderate	Low	Low	Low	Low	Low	Moderate
Kalem [27]	Serious	Moderate	Low	Low	Low	Low	Moderate	Serious
Sabater-Martos [28]	Moderate	Low	Low	Low	Low	Low	Low	Moderate
Wirtz [29]	Serious	Moderate	Low	Low	Low	Low	Low	Serious

### Patient-reported outcome measures

Four studies reported on post-operative PROMs using four different scales (Table 5). Due to heterogeneity of constructs measured, study design, and one study describing categorical results, PROMs were not pooled and synthesised quantitatively using standardised mean difference [31]. Overall, there was weak evidence of a slightly favourable effect of AW on PROMs. However, the small mean differences were likely not clinically significant, and overlapping confidence intervals would suggest no real difference in effect estimates.

**Table 5:** Patient-reported outcome measures at latest post-operative follow-up, reported in four of the included studies

Study	Functional outcome assessed	Scale descriptor	Reported result		Comments
			AW	OW	
RCT					
Peres [23]	Lysholm Knee Scoring Scale (LKSS)	Assesses eight clinical domains, producing an overall score between 0 and 100 (< 65 poor, 65–83 fair, 84–94 good, 95–100 excellent) [31]	93.8^¶^ ± 2.3 — equates to ‘good’ score	87.2^¶^ ± 5.5 — equates to ‘good’ score	Difference non-significant
Cohort study					
Balabaud [24]	Bussiere and Beaufils functional scale (BBFS)	Considers patient-reported pain and ROM and is reported as an ordinal scale (excellent, good, fair, poor) [32]	‘Good’ in 15/21 knees	‘Good’ in 4/19 knees	Difference non-significant
Sabater-Martos [28]	Western Ontario and McMaster Universities Osteoarthritis Index (WOMAC)	Considers pain (0–20), stiffness (0–8) and functional impairment (0–68), with lower scores reflecting superior states [33]	17^¶^ ± 15.4—equates to ‘slight difficulties’	16.1^¶^ ± 15.9—equates to ‘slight difficulties’	Difference non-significant
Wirtz [29]	Larson score	Considers seven clinical domains (pain, walking, function, ROM, strength, flexion contracture, laxity), producing a score between 0 and 100 (< 60 poor, 60–69 fair, 70–84 good, 85–100 excellent) [34]	74^¶^ ± 17.5—equates to ‘good’ score	61^¶^ ± 18.5—equates to ‘fair’ score	Difference only significant if operated on within 5 days of symptom onset (favours AW)

^¶^Mean value ± standard deviation (range)

### Range of movement

**Fig. 2:**
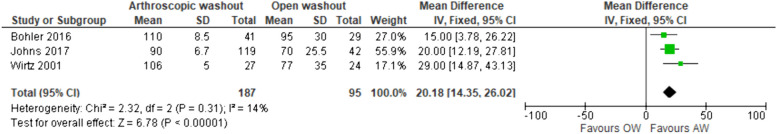
Forest plot of the comparison of AW and OW for post-operative ROM

Four cohort studies described ROM at latest follow-up (Table 6). The mean difference in ROM was 20.18° (95% *CI* 14.35, 26.02; *p* < 0.00001), favouring AW (Fig. 2). No significant heterogeneity was observed (*I*^2^ = 14%). Findings by Kalem et al. [27] were excluded from this meta-analysis, as necessary information regarding the interquartile range was neither reported nor provided when requested from the corresponding author.

**Table 6:** Range of movement at latest post-operative follow-up, reported in four of the included studies

Study	Reported result		Comments
	AW	OW	
Böhler [25]	110 ± 8.5^¶^	95 ± 30^¶^	Difference reaches statistical significance (*p* < 0.001)
Johns [26]	90 ± 6.7^¶^	70 ± 25.5^¶^	Difference reaches statistical significance (*p* = 0.016)
Kalem [27]	100^¥^	100^¥^	Difference non-significant
Wirtz [29]	106 ± 5^¶^	77 ± 35^¶^	Difference significant if operated on within 5 days of symptom onset

^¶^Mean value ± standard deviation. ^¥^Median value

On age-adjusted subgroup analysis, Böhler et al. [25] found the difference in mean ROM between AW and OW groups persisted (*p* = 0.008).

### Secondary outcomes

Table 7 details the requirements for re-operation and whether synovectomy was performed during the index procedure. Re-operation was necessary in 31.7% (77/243) of patients following AW and 33.8% (51/151) of patients following OW. Practice regarding synovectomy varied. Owing to inconsistency in reporting, we were unable to look for association between synovectomy and re-operation requirements.

**Table 7:** Practice regarding synovectomy and return to theatre after index procedure, described in each study

Study	Synovectomy at index procedure		Further procedure				Comment
	AW	OW	AW	Timeframe; details	OW	Timeframe; details	
RCT							
Peres [23]	10 (100%)	11 (100%)	0	-	2 (18.2%)	Within 7 days; 2 OW	Difference non-significant
Cohort study							
Balabaud [24]	1 (4.8%) If severe swelling/effusion	13 (68.4%)	6 (28.6%)	‘Early’ (not specified) 5 OW/synovectomy, 1 open arthrolysis	3 (15.8%)	‘Early’ (not specified) 1 OW, 1 OW/synovectomy, 1 arthrodesis	-
Böhler [25]	Not specified (done at surgeon’s discretion)	Not specified (done at surgeon’s discretion)	2 (4.9%)	Median 3.0 days (within 3 months); no details of procedure	6 (20.7%)	Median 3.0 days (within 3 months); no details of procedure	Higher following OW (*p* = 0.041)
Johns [26]	-		60 (50.4%)	Timeframe not specified; 51 AW, 9 OW	30 (71.4%)	Timeframe not specified; 2 AW, 28 OW	Higher following OW (*p* = 0.02)
Kalem [27]	-		2 (15.4%)	Timeframe not specified (within 6 months) No details of procedure	4 (36.4%)	Timeframe not specified (within 6 months); no details of procedure	-
Sabater-Martos [28]	12 (100%)	15 (100%)	5 (42.7%)	Timeframe not specified No details of procedure	2 (13.3%)	Timeframe not specified No details of procedure	-
Wirtz [29]	27 (100%)	-	2 (7.4%)	Timeframe not specified; 2 AW	4 (16.7%)	Timeframe not specified; 1 OW, 3 arthrodesis	-

As the timeframe from index to second procedure was often not specified, we were unable to report 30- and 90-day re-operation rates. The second procedure typically paralleled the index; 53 AW patients (68.5%) underwent further AW, and 33 OW patients (64.7%) underwent further OW. The nature of subsequent procedure(s) was not specified for 21 patients.

Additionally, it was often not reported whether single or multiple repeat procedures were necessary. Johns et al. [26] reported that fewer irrigation procedures were required following AW (1.79 ± 0.96) than following OW (2.42 ± 1.5) (*p* = 0.010).

**Fig. 3:**
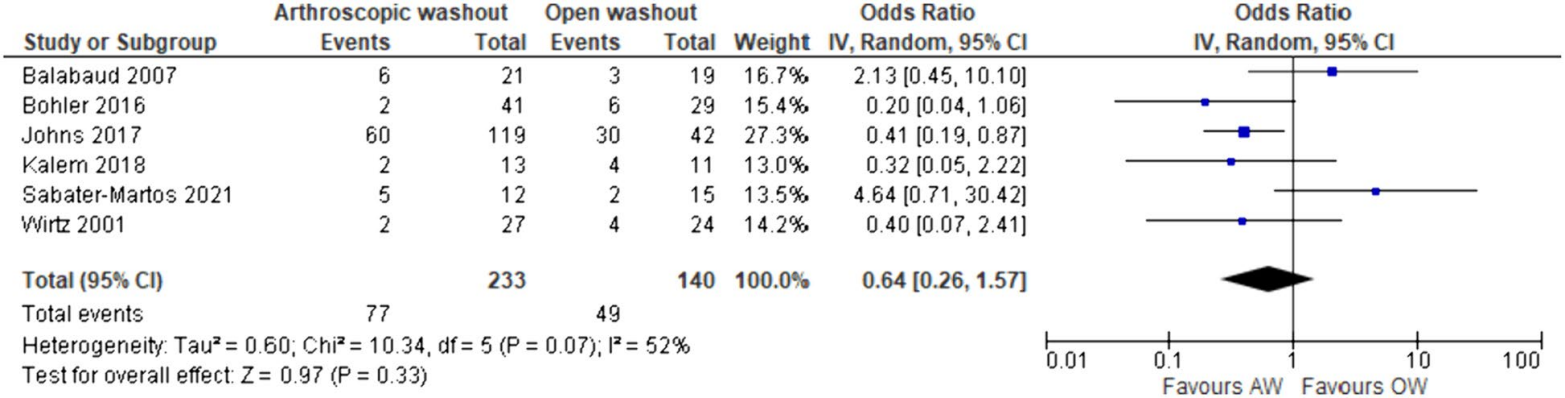
Forest plot of the comparison of arthroscopic washout and open washout regarding need for further washout

Meta-analysis of the six cohort studies suggested a tendency for lower re-operation requirement following AW (*OR* 0.64, 95% *CI* 0.26–1.57, *p* = 0.44) (Fig. 3). Moderate interstudy heterogeneity was observed (*I*^2^ = 52%). Data from Peres et al. [23] were not included in this model owing to the difference in study design; however, the authors reported no difference in effectiveness of treatment.

After age adjustment, Böhler [25] found the difference in re-operation requirements between groups persisted (*p* = 0.008). Similarly, Johns [26] found that the superiority of AW persisted after adjustment for age, sex, comorbidity, and positive joint culture (*OR* 2.56, 95% *CI* 1.1, 5.9; *p* = 0.027).

### Quality of evidence

The summary of the GRADE assessment [19] for each outcome is outlined in Table 8. The evidence certainty ranged from moderate to very low for all outcomes assessed in this systematic review. This was mostly because of starting with a low rating because the data were mostly from observational studies, and the certainty of the evidence was further downgraded for risk of bias or inconsistency.

**Table 8:** Summary of findings for arthroscopic washout compared with open washout for native knee joint septic arthritis

Patients or population: Adult patients with septic arthritis of the native knee joint Intervention: Arthroscopic washout (AW) Comparison: Open arthrotomy washout (OW)						
Outcomes	Illustrative comparative risks		Relative effect	Number of participants (studies)	Certainty of the evidence (GRADE)	Comments
	AW	OW				
Lysholm Knee Scoring Scale [32]	The mean score was 93.8 points	The mean score was 6.6 points lower	-	21 (1 RCT)	⨁⨁⨁O^1^	AW may result in a superior LKSS, however, difference non-significant
Bussiere and Beaufils functional scale [33]	Function regarded as ‘good’ in 71.4%	Function regarded as ‘good’ in 21.1%	-	40 (1 cohort study)	⨁⨁OO^2^	AW may result in a superior BBFs, however, difference non-significant
Western Ontario and McMaster Universities Osteoarthritis Index [34]	The mean score was 17 points	The mean score was 0.9 points lower	-	27 (1 cohort study)	⨁⨁OO^3^	AW may result in a superior WOMAC, however, difference non-significant
Larson score [35]	The mean score was 74 points	The mean score was 13 points lower	-	51 (1 cohort study)	⨁OOO^4^	AW may result in a superior Larson score, however, difference non-significant
Range of movement	The mean ranged from 90 to 106°	The mean ranged from 70 to 95°	Mean difference 20.18°	282 (4 cohort studies)	⨁OOO^5^	AW associated with superior post-operative ROM
Need for further intervention	Rates of reoperation ranged from 4.0 to 50.4% in the observational studies, 0 in the RCT	Rates of reoperation ranged from 13.3 to 71.4% in the observational studies, 18.2% in the RCT	*OR* 0.64	384 (6 cohort studies, 1 RCT)	⨁OOO^6^	AW may be associated with lower re-operation requirement, however, difference non-significant

GRADE Working Group grades of evidence [19]. ⨁⨁⨁⨁High certainty, we are very confident that the true effect lies close to that of the estimate of the effect. ⨁⨁⨁OModerate certainty, we are moderately confident in the effect estimate: the true effect is likely to be close to the estimate of the effect, but there is a possibility that it is substantially different. ⨁⨁OOLow certainty, our confidence in the effect estimate is limited: the true effect may be substantially different from the estimate of the effect. ⨁OOOVery low certainty, we have very little confidence in the effect estimate: the true effect is likely to be substantially different from the estimate of effect. Explanations: 1, Down-graded one level due to some concerns regarding bias in measurement of outcomes; 2, single observational study with possible bias due to confounding and selection bias; 3, single observational study with moderate risk of bias due to confounding; 4, single observational study down-graded 1 point due to at high risk of bias due to confounding and moderate risk of selection and reporting bias; 5, down-graded 1 point due to high risk of confounding in several of the included observational studies; no significant heterogeneity between studies (*I*^2^ 14%); 6, down-graded 1 point due to high risk due to confounding in several of the included observational studies; moderate heterogeneity present (*I*^2^ 52%)

### Publication bias

We were unable to undertake Egger’s test for publication bias, as Egger’s test has insufficient power to distinguish chance from real funnel plot asymmetry with fewer than 10 studies [36].

## Discussion

Septic arthritis of the native knee can be joint- and life-threatening; thus, prompt, effective management is paramount. Our findings suggest that AW has a tendency for favourable functional outcomes and re-operation rates compared with OW. However, the evidence is uncertain due to moderate-serious risk of bias and inter-study heterogeneity.

### Comparison with other studies

The present study represents the first systematic review focusing primarily on function following AW and OW. Our findings agree with PROMs and ROM described in reviews by Panjwani [14] and Liang [13], respectively. This was predictable, as we retrieved just one additional study reporting PROMs [28], and none further reporting ROM. Findings by Kalem [27], which did not show a difference regarding ROM, were not included in the meta-analysis because information required for pooling of the data was not provided.

Our findings suggested that AW may be associated with lower re-operation rates, given the direction and magnitude of the risk estimate (*OR* 0.64). However, the confidence intervals were imprecise suggesting heterogeneity, so the results should be interpreted cautiously. In keeping with our findings, Liang [13] showed a possible trend for lower rates of reinfection following AW (*OR* = 0.85; *p* = 0.44), whilst Panjwani [14] reported substantially reduced risk of reoperation (*RR* = 0.69; *p* = 0.0006). Both these reviews included additional studies in their pooled analyses which were excluded from the present study due to omission of PROMs [37–41]. Additionally, Panjwani [14] combined effect estimates from randomised and non-randomised studies, which is generally inappropriate [42].

### Explanation of findings

It could be suggested that the less-invasive AW is associated with superior post-operative function, owing to smaller surgical incisions and shorter post-operative recovery. The reported difference in mean ROM (20.18°) is likely highly clinically significant; whilst not previously studied in the septic arthritis setting, in the setting of stroke, the minimum clinically important difference (MCID) was under 10° [43]. However, given the observational nature of six of the included studies, there is the risk of confounding. We noted that patients with higher Gächter-stage disease [24, 25, 29], mean preoperative temperature [23], and more risk factors for SA development [26] were selected for OW. Such preference for OW in higher Gächter-stage disease has been described elsewhere [44]. Thus, poorer functional outcomes might be expected.

Requirement for reoperation may be confounded by patient factors, including Gächter stage, pyrexia at presentation, body mass index > 45 kg/m^2^, elevated inflammatory markers, and immunosuppression [5, 44–46]. Of the included studies, only Böhler [25] and Johns [26] adjusted for confounders. Similarly, re-operation requirement may be influenced by intervention factors, including time from presentation to index and subsequent procedures, total number of procedures, and individual surgeons’ thresholds for synovectomy and reoperation. Due to inconsistent reporting, we were unable to stratify or adjust for these factors which may have affected outcomes in the pooled analysis; thus, one should interpret these results with caution.

It is also possible that the observed associations may have arisen due to underpowering, as no prior sample size calculation was undertaken. Post hoc analysis suggested adequate power in the RCT [23] and two cohort studies [25, 26]; however, this may not be the case for the remaining studies.

### Implications of findings

We suggest that AW is acceptable to patients and efficacious in the treatment of native knee SA and thus should be routinely used in the management of this condition. We have presented strong evidence in favour of AW regarding ROM and weak evidence regarding PROMs.

As AW was associated with a tendency for reduced re-operation requirement, this may decrease healthcare costs, and we suggest further investigation is warranted. We note, however, that none of the studies utilised a generic health-related quality-of-life assessment tool, which has been recommended to be used in combination with condition-specific scales to facilitate economic assessment [47].

Additionally, the use of four different PROMs scales has rendered direct comparison between studies impossible, and with the data provided, it was not possible to calculate standardised mean difference. Of the scales used, only the BBFS has been described in SA of the native knee [33], and the MCID has been established in the SA context for none of the scales. Such use of unvalidated tools may render results less reliable, and differences observed may not be clinically relevant. Furthermore, it is possible that the scales used do not actually reflect what is pertinent to the patient population; to our knowledge, this has not been explored qualitatively. Additionally, we have considered PROMs and ROM at latest follow-up. As average follow-up duration varied, and it was often unclear when measurements were obtained, these functional results may not be directly comparable.

### Strengths and limitations

A robust search of multiple databases and rigorous approach to study selection was employed. This ensured that all available relevant citations were identified and outcomes extracted. However, owing to the lack of high-quality studies, the findings should be interpreted with caution. Limitations of the six cohort studies include their retrospective nature, typically small sample sizes, and no blinding of outcome assessors, whilst the small, single-centre nature of the RCT may limit the external validity of their findings. Furthermore, owing to the small number of studies included, we were unable to test for publication bias.

As SA represents an increasing clinical concern, a definitive RCT is warranted. In contrast to the RCT by Peres [23], this should be multicentre and with prior sample size calculation, in order to improve external validity and ensure sufficient power to capture the outcomes of interest. Subgroup analysis may also wish to consider the appropriateness for AW or OW by disease severity, association between disease severity and functional outcomes, and the role of synovectomy at initial washout. Despite RCTs being the gold standard for clinical research, their use in assessing the effectiveness of orthopaedic interventions has limitations. They are labour intensive, expensive, and need large sample sizes. Real-world evidence, such as nesting analysis within arthroplasty registries, may represent better investigative avenues.

## Conclusion

Based on the available evidence, we conclude that AW results in favourable post-operative ROM, similar PROMs, and a tendency for lower re-operation rates compared with OW. Thus, AW is acceptable for use in the treatment of native knee SA. However, as OW tended to be used in those with more severe disease, there may be confounding by indication. Therefore, there is no evidence to suggest that OW should not be used, for example, should arthroscopic treatment be unavailable.

Despite SA being a growing area of clinical concern, higher-quality evidence is lacking. Clinical and methodological heterogeneity of the included studies limits one’s ability to make meaningful comparisons. This systematic review highlights the need for more definitive large clinical trials, with a particular focus on patient-reported and functional outcomes.
